# HIV awareness campaigns, Knowledge and practices among pregnant women living with HIV in northern Uganda

**DOI:** 10.11604/pamj-oh.2023.11.10.39159

**Published:** 2023-06-30

**Authors:** Jonathan Ogena, Didan Jacob Opii, Betty Nakku, Cindrella Aceng, Tonny Oola, Veronica Kobusinge, Godfrey Zari Rukundo, Anna Grace Auma

**Affiliations:** 1 Lira University, P.O.BOX 1035, Faculty of Health Sciences Lira University, Lira, Ouganda; 2 Department of Psychiatry, Mbarara University of Science and Technology, Mbarara, Ouganda

**Keywords:** HIV, awareness, campaigns, knowledge, pregnant women

## Abstract

**Introduction:**

despite significant progress made in HIV care and research, there are still many misconceptions on acquisition, treatment and progress of HIV especially in rural communities. Different strategies have been utilized to spread reliable knowledge to different audiences. One of the strategies has been the use of community awareness campaigns. However, it is not clear if these campaigns have been effective and if they reached the different sub-populations in the community. This study aimed to assess the knowledge and practices of HIV community awareness campaigns and associated factors among pregnant women living with HIV in Oyam district in northern Uganda.

**Methods:**

it was a quantitative cross-sectional study in a consecutively selected sample of 416 pregnant women living with HIV. An interviewer-administered questionnaire was used to collect data on awareness, sources of information, content of the messages, utilization of the awareness messages and the perceptions about the campaigns. In addition, we determined factors associated with knowledge about the community campaigns. The level of knowledge was determined by the participant' response indicating whether they had prior knowledge about awareness campaigns or not. Data were analyzed using SPSS version 23 using Chi-square and logistic regression at 95% confidence interval and a p-value of 0.05 for statistical significance.

**Results:**

of the 416 participants in the study, 92.5% (n=385) had prior knowledge about HIV awareness campaigns and 97.6% (n=406) had specifically heard about anti-HIV-related stigma campaigns. The most common sources of information were radio (43.3%), health education sessions at health facilities (44%), and family members (2.9%). The received information was on HIV transmission, antiretroviral therapy, HIV related stigma as well the effect of stigma on self-esteem, hopelessness, HIV related abuse and ART adherence. Knowledge was associated with having a source of income (OR= 0.162, 95%CI 0.034-0.775, P= 0.023), having heard about HIV-related Stigma (OR=0.051, 95%CI 0.003-.949, P= 0.046), availability of community linkage facilitators (OR= 0.077, 95% CI 0.011-0.537, P= 0.010), use of awareness messages by community members (OR= 13.887, 95% CI 1.316-146.6, P=0.029) and the source of HIV awareness information (OR= 0.462, 95% CI 0.237-.902, P=0.024).

**Conclusion:**

although there is still high HIV-related stigma in general public, there is increased awareness among pregnant women. Availability of community linkage facilitators and use of appropriate sources of information seems to be helping with increasing community knowledge about HIV awareness campaigns.

## Introduction

There have been many interventions in place for HIV care but 25% of people living with HIV/AIDS still do not access HIV treatment [1]. This challenge is worse in settings where the HIV burden is highest including sub-Saharan Africa. Several factors are associated with limited access to HIV care. These include stigma and discrimination, staff shortages and lack of sustained supply of medications [2]. Different strategies have to be put in place to address each of the barriers to care In Uganda, in order to improve access to HIV care services, the Ministry of Health has continued to rally different organizations to establish country-wide social-behavioral interventions such as knowledge sharing which is implemented as HIV awareness campaigns, capacity building, advocacy at policy level and research about HIV related stigma [3].

HIV awareness campaign is a means of sensitization to the community in matters related to HIV and its effects. This information includes modes of transmission, prevention strategies, treatment access, and stigma related to the infection [4]. Awareness has several definitions and is sometimes used interchangeably with knowledge. In this study, awareness was defined as a knowledge of, or perception of a situation or fact, feeling, or being conscious of events, objects, thoughts, emotions, or sensory patterns. The level of awareness was assessed by with a question requiring a 'yes' or 'no' answer. The question was: have you ever heard about HIV awareness campaigns? The awareness campaign has been widely implemented across countries in an attempt to reduce HIV-related stigma and it has shown positive impact [5]. However, there is limited information on the level of awareness in rural community settings. This study aimed to assess the level of awareness and practices HIV awareness campaigns and associated factors among pregnant women living with HIV in Oyam District in northern Uganda. The research questions addressed by study were: 1) What is the extent of uptake of HIV awareness campaigns towards HIV related stigma and discrimination among HIV positive pregnant women in attending Antiretroviral (ART) clinic at Aber Hospital, Anyeke HC IV, Iceme HC III and Ngai HC III? 2) What are the factors associated with uptake of HIV awareness campaigns towards HIV related stigma and discrimination among HIV positive pregnant women in attending ART clinic?

## Methods

### Study setting and study population

The study was conducted among pregnant women living with HIV and attending ART clinics at Aber Hospital, Anyeke HC IV and, Agulurude HC III in Oyam district in northern Uganda in February-April 2020. Oyam District is about 260 kilometers from the capital city of Kampala. Oyam is bordered by Gulu District to the North, Kiryandongo District to the southeast, Kole District to the East, Apac District to the South and Nwoya District to the West. Aber Hospital has an average daily clientele of about 70 HIV positive clients, of whom about 10 are pregnant women linked from antenatal clinic to the ART clinic for treatment and care.

### Study design

This was a quantitative cross-sectional study using a questionnaire that collected data on awareness, sources of information, content of the messages, utilization of the awareness messages, interest to learn more as well as perceptions about the campaigns. In addition, we determined factors associated with awareness.

### Sample size and sampling

The sample for the study was calculated using the Kish-Leslie (1965) formula with a proportion of 50%, Z=1.96, and a precision of 5%. The proportion of 50% was used because there was no known proportion for HIV positive pregnant women, this yielded a maximum sample size of 416 after adjusting with a 10% non-response rate [6]. The precision of 5% also generates a power (1-β) of 80%.

### Procedure for data collection

Before starting data collection, we engaged staff at the respective health centers to help us identify the potential participants. Consecutive sampling was used to select participants on the days they came to the ART clinics at the respective health facilities until we achieved the target sample size. An interviewer-administered structured questionnaire was completed by 416 pregnant women living with HIV in February-April 2020. The questionnaire was pretested among 42 HIV-positive pregnant women at Amach HC IV in the neighboring district of Lira. Three research assistants were trained on the study purpose of the study and the administration of the questionnaire. Data were collected on participant employment, education level, source of income, awareness on anti-HIV-related campaigns and the factors associated awareness such as source of communication of information presence of community linkage facilitators, community opinion on HIV related stigma and the willingness to learn more about HIV awareness messages.

Every HIV-positive pregnant woman attending the ART clinic in the study period was contacted and asked to participate in the study. Written informed consent was obtained from every participant. Each participant retained a copy of the consent form for reference purposes. Pregnant women living HIV and aged less than 18 years were considered emancipated minors and allowed to provide informed consent. Those who were too sick and to complete the questionnaire were excluded from the study. The questionnaire took about 10-15 minutes to complete and the participants were given their transport refund. The data collected from participants were on sociodemographic characteristics such as age, education, employment status and sources of income. We also collected data on predictors of factors associated with use of community awareness campaigns such as mode of communication with the clients (media, radio, health education talks), kind of information being communicated, interest to learn more, and perceptions about the predictors of uptake of HIV awareness campaigns.

### Data management and analysis

Data collected were checked daily for completeness. The questionnaires were coded and the de-identified data were entered into the SPSS software. The questionnaires were kept under key and lock to ensure confidentiality. Data were analyzed using SPSS version 23.0. Awareness of HIV awareness campaigns was determined using univariable analysis as a proportion. It was a dichotomous variable with a 'yes/no' answer. Continuous variables were analyzed using mean and standard deviation, and frequencies were used for categorical variables. Bivariable analysis was done to find associations with the outcome variable (knowledge about HIV awareness campaigns). During data analysis, we assumed that the level of awareness was influenced by several factors including sociodemographic characteristics, social of information, and influence of community linkage facilitators. We also assumed that the factors would influence each other. Therefore, we needed to know the factors that would independently be associated with the level of awareness. Odds ratios and the corresponding 95% confidence intervals were used to measure the associations of statistical significance. All variables with p-values < 0.2 at bivariable analysis were entered into a multivariable logistic regression model to determine independent associations with of the knowledge of HIV awareness campaigns. The model fitness was checked using the Hosmer Lemeshow test at P>0.05. The analysis aimed at answering the following questions: 1) what were the characteristics of the pregnant women living with HIV and receiving HIV care at selected health care facilities in Oyam District in northern Uganda? 2) What factors were associated with the level of knowledge about HIV awareness campaigns among pregnant women living with HIV and receiving HIV care at selected health care facilities in Oyam District in northern Uganda? 3) What factors were independently associated with the level of knowledge about HIV awareness campaigns among pregnant women living with HIV and receiving HIV care at selected health care facilities in Oyam District in northern Uganda?

### Ethical considerations

Approval was given by Gulu University Research and Ethics Committee (GUREC): reference number GUREC-2021-39 and the National Council for Science and technology. Clearance was obtained from the District Health Officer Oyam District and the office of the Health facility in charge to carry out the study from the facility. The study participants were informed about the purpose, benefits, and risks of participating in the study and written informed consent was obtained from the participants. Confidentiality and privacy were maintained by using codes instead of participants' names and interviewing participants one at a time in a private space. Pregnant teenagers below 18 years of age were treated as emancipated minors.

### Funding

Research reported in this publication was supported by the Fogarty International Center (the U.S, Department of State's Office of the U.S. Global AIDS Coordinator and Health Diplomacy and the President's Emergency Plan for AIDS Relief) of the National Institutes of Health. Grant award Number R25TW011210. The content is solely the responsibility of the authors and does not necessarily represent the official views of the National Institute of Health.

### Ethics approval and consent to participate

All methods were performed in accordance with the relevant guidelines and regulations. The study was reviewed and approved by the Gulu University Research Ethics Committee (GUREC-2021-39). Additional permission was sought from the health facility in-charges. Before participating in the study, we obtained written informed consent from the adult participants. The investigators and research assistants underwent training in responsible conduct of research and understood the importance of participant privacy, confidentiality and autonomy. At data entry participant identifiers (such as names, phone contacts) were included.

## Results

### Participant socio-demographic characteristics

A total of 416 pregnant women with mean age of 27.85 (S.D= 6.62) participated in the study (Table 1). The majority (51.9%) was in the age range of 25-34 Years, and more than a third (35.1%) of them had no formal education. Most of them did not have formal employment (87.3%) but had some form of income (71.9%).

### Factors associated with HIV community awareness campaigns

About 92.5% (n=385) of the participants had prior knowledge about HIV awareness campaigns and 97.6% (n=406) had specifically heard about anti-HIV-related stigma campaigns. The level of awareness about HIV related stigma awareness campaigns was determined by the participant response indicating whether they had prior knowledge about awareness campaigns. We also considered the mode of communication, information communicated, their interest to learn more, perceptions about the campaigns, as well as the HIV related myths and misconceptions.

#### Mode of communication:

the participants reported different sources of information. The most common sources were radio (43.3%) health education sessions (44%) at the health facilities and family members (2.9%). These sources of information were the most preferred; health facility talks (46.4%), radio (42.3%) and family members (2.4%).

#### Information communicated:

much of the received information was on HIV related stigma with a focus on low self-esteem (23.3%), effect of stigma on hopelessness (14.2%), HIV related abuse (20.4%) and ART adherence (9.6%).

#### Interest to learn more:

according to this study, 89.9% had attended previous awareness campaign meetings. The most preferred modes of campaigns were radio (44.5%), drama (26.9%) and health facility education (13.2%). Only 8.2% (n=34) had not attended any previous awareness campaigns and almost all (99.5%) would be glad to attend another awareness session if they were given opportunity.

#### Perceptions about the campaigns:

according to the participants, most community members (87.7%) don't use the HIV awareness messages (Table 2).

### Predictors of uptake of HIV awareness campaign

Multivariate analysis was performed to determine the factors which were independently associated with the awareness of HIV awareness campaigns. All variables with a p-value < 0.2 at bivariable analysis were entered in a multivariable logistic regression model as independent variables with awareness as the outcome variable. Table 3 shows that the predictors of having knowledge about HIV awareness campaigns were: having a source of income (OR= 0.162, 95%CI 0.034-0.775, P= 0.023), having heard about HIV-related stigma (OR=0.051, 95%CI 0.003-.949, P= 0.046), availability of community linkage facilitators (OR= 0.077, 95% CI 0.011-0.537, P= 0.010), community members' use of awareness messages (OR= 13.887, 95% CI 1.316-146.6, P=0.029) and the source of HIV awareness information (OR= 0.462, 95% CI 0.237-.902, P=0.024).

## Discussion

This study aimed to assess the knowledge and practices of HIV awareness campaigns and associated factors among pregnant women living with HIV in Oyam District in northern Uganda. We found that most of the pregnant had prior knowledge about HIV awareness campaigns and had heard about anti-HIV-related stigma campaigns. The predictors of knowledge were: having a source of income, having heard about HIV-related stigma, availability of community linkage facilitators and the source of HIV awareness information.

### Knowledge about HIV awareness campaigns

The high level of knowledge about HIV campaigns is a good indicator that community awareness strategies are getting to the intended audience. This is similar to what has been described in previous studies in Uganda [7,8] and the Philippines [9,10]. There is generally fair dissemination of information to various rural communities in various countries [11]. This study was conducted in health facilities. It is possible that community based studies may find different results.

### Source of income and level of knowledge on awareness campaigns

In our study, we found that having a source of income was associated with having knowledge on HIV awareness campaigns. This could be due to other related characteristics like acquisition of telephone, radio, newspapers and ability to move to places where information can be obtained. Having a source of income is also associated with opportunities for higher education and decision making [12].

### HIV-related stigma and awareness campaigns

The findings of this study indicate that prior knowledge on HIV-related stigma are significantly associated with uptake of HIV awareness campaigns, this could be explained by the theory of frequent exposure to information being more likely to influence the use of information, these study findings are consistent with findings from a study conducted in Ghana by Fenny *et al.* in 2017, which found that sound knowledge on HIV is critical in behavior change which indicates acceptance and use of the information [13]. Other studies examining theories of behavior change agree with this study that a person will take time to accept the information and use it depending on how long they have had exposure to the messages and how clear the messages are conveyed to them [14]. The similarity in the findings could be in the similar study tools used, an interviewer-administered questionnaire, and similar study populations.

### Community linkage facilitators and HIV awareness campaigns

In our study, we found that the presence of community linkage facilitators was statistically associated with knowledge about HIV awareness campaigns. These community linkage facilitators have the roles of educating community members on matters related to HIV prevention and treatment, aiding linkage of HIV positive clients to the health facility for treatment and reducing HIV-related stigma in the community. Our finding implies that the community linkage facilitators are very important for increasing HIV awareness in communities. This is similar to a study by Gulaid, in 2015, found out that to have a successful saturation of the community we need to involve the community [15] because the people who live in that community and are familiar with the cultures and beliefs of a particular community and are in a better place to bridge the gap of information giving, access to HIV services and utilization of HIV related information [9]. However, this study used a large study population involving literature reviews, stakeholder consultations, and countrywide visits. Comparatively, a study by Nieto in 2019, in Tanzania found that community linkage facilitators improved the linkage of clients and increased information on HIV-related stigma [12-14]. This was a qualitative study that was able to explore more from the community, compared to our study these two studies used different study methods but had similar findings. This implies that community linkage facilitators are vital in increasing information on HIV awareness and reduction of HIV-related stigma.

### Source of information on HIV awareness

The most common and most preferred sources of information about awareness were radio, health facility talks and family members. This finding indicates that what is required is readily available and inexpensive. Most communities have access to radios using locally spoken languages. These radios often offer free airtime for health related messages. In addition, health education talks are often part of routine care at most health facilities and do not require extra expenditure. Our findings are similar to what has been reported in previous studies about the importance of social media. However, our findings are contrary to what has been reported by Bekalu and Eggermont (2014) who indicated that mass media campaigns were not helpful for the rural communities [16]. The difference could be due to the technological progress in the past eight years in which more radios and technology have reached rural communities. In addition, this study was conducted among pregnant women who are expected to have many visits to health facilities for pregnancy checks. This exposes them to extra information compared to other community members. It is possible that the findings would be different among non-pregnant women living in the same communities.

### Implications for HIV care

There is need to emphasize involvement of community linkage facilitators in HIV care so that they can act as change agents in their communities. Radio communication and health facility talks should be strengthened since they are currently showing benefits.

### Study limitations

This study was conducted among pregnant women who were already HIV positive with regular attendance of health facilities. Their views may not represent those who are HIV negative and not pregnant. This study was done in a rural setting and the findings may not be generalized to the urban settings in Uganda.

## Conclusion

Although there is still high HIV-related stigma in general public, most of the pregnant women living with HIV have a high level of knowledge about HIV awareness campaigns. Availability of community linkages and the information given during antenatal visits could be responsible for the high level of awareness about anti-HIV related stigma. Interventions targeting the reduction of HIV-related stigma and discrimination should factor in the identified factors and strengthen their implementation for positive impact in the community.

## Figures and Tables

**Table 1:** socio-demographics characteristics of HIV-positive pregnant women in Oyam district, northern Uganda, April 2021 (N=416)

Variable	Description	Frequency n (%)
Age (years)	15-24	139(33.4)
	25-34	216(51.9)
	35 and above	61(14.7)
Education	No formal education	146 (35.1)
	Completed primary	184(44.2)
	Completed secondary	57(13.5)
	Tertiary education	29 (6.9)
Employment status	Unemployed	363 (87.3)
	Employed	53 (12.7)
Any source of income	No source of income	117 (28.1)
	Has some source of income	299 (71.9)

**Table 2:** factors associated with awareness and perceptions about HIV awareness campaigns among women in Oyam district, northern Uganda, April 2021 (N=416)

Variable	Description	Frequency (%)	P-Value
Age (years)	15-24	139(33.4)	0.000
	25-34	216(51.9)	
	35 and above	61(14.7)	
Employment status	Unemployed	363 (87.3)	0.073
	Employed	53 (12.7)	
Level of education	No formal education	146 (35.1)	0.008
	Primary	184(44.2)	
	Secondary/ Tertiary	86(20.7)	
Having a source of income	No source of income	117 (28.1)	0.000
	Has source of income	299 (71.9)	
Source of information on HIV awareness campaigns	Radio	180 (43.3)	0.000
	Health facility	183 (44)	
	Family	12 (2.9)	
	Other sources	41 (9.9)	
Availability of community linkage facilitators	Yes	376 (90.4)	0.000
	No	40 (9.6)	
wanting to learn more about HIV awareness campaigns	Yes	414 (99.5)	0.856
	No	2 (0.5)	
Do you think community members use of awareness messages	Yes	51	0.000
	No	365	
Previous attendance of awareness meetings	Yes	374 (89.9)	0.000
	No	42 (10.1)	
Having heard about HIV related stigma and discrimination	Yes	406 (97.6)	0.000
	No	10 (2.4)	

* P<0.05 Generated by Chi square test was used to assess the association between the various independent variables and the outcome variable (level of awareness).

**Table 3:** predictors of the awareness HIV awareness campaigns among pregnant women living with HIV in Oyam district, northern Uganda, April 2021 (N=416)

Variable	Description	cOR (95%CI)	Adjusted odds ratio (aOR) 95% C.I.
Age (years)	15-24 25-34 35 and above	3.796 (1.895-7.608)	0.802 (0.266-2.420)
Having a source of income	No Yes	0.250 (0.118-0.529)	0.162 (0.034-0.775)
Level of education	No formal education Primary Secondary and Tertiary	2.551 (1.402-4.639)	1.938 (0.178-21.109) 1.216 (0.118-12.575)
Ever heard about HIV Stigma and discrimination	No Yes	0.015 (0.003-0.075)	0.051 (0.003-.949)
Attendance of campaign meetings	No Yes	119.925 (40.723-353.164	0.464 (0.037-5.747)
Availability of community facilitators	No Yes	62.235 (24.311-159.322)	0.077 (0.011-.537)
Use of awareness messages by community members	No Yes	0.010 (0.003-0.030)	13.887 (1.316-146.6)
Information source	Radio Health facility Family Other sources	0.241 (0.165-0.354)	0.462 (0.237-.902)

* The tests assess the association between the knowledge about HIV awareness campaigns and the various factors.

## What is known about this topic

- Use of media for community awareness campaigns is effective;
- Community awareness campaigns lead to HIV stigma reduction;
- Lack of awareness leads to low self-esteem, hopelessness and ill health.

## What this study adds

- There is a high level of knowledge among pregnant women living with HIV concerning HIV awareness campaigns;
- Access to antenatal services and health education talks are associated with high knowledge about HIV awareness campaigns;
- Presence of community linkage facilitators has an impact on the level of awareness among pregnant women living with HIV.
